# Comparing Tubeless and Tubed Approaches in Percutaneous Nephrolithotomy for Moderate Renal Calculi: Outcomes on Safety, Efficacy, Pain Management, Recovery Time, and Cost-Effectiveness

**DOI:** 10.7759/cureus.39211

**Published:** 2023-05-19

**Authors:** Surag KR, Anshuman Singh, Pritam Sharma, Vivek Pai, Anupam Choudhary, Shreenath Patil

**Affiliations:** 1 Urology, Kasturba Medical College, Manipal, IND; 2 Urology, A.J. Institute of Medical Sciences, Mangalore, IND

**Keywords:** bleeding complications, tubeless pcnl, tubed pcnl, percutaneous nephrolithotomy (pcnl), urolithiasis

## Abstract

Introduction

This study focuses on investigating the effect of routine nephrostomy tube placement in patients with moderate renal calculi of size 2.5 cm or less who undergo uncomplicated percutaneous nephrolithotomy (PCNL) procedures. Previous studies have not specified whether only uncomplicated cases were included in the analysis, which may affect the results. This study aims to provide a clearer understanding of the effect of routine nephrostomy tube placement on blood loss in a more homogeneous patient population.

Materials and methods

A prospective randomized controlled trial (RCT) was conducted at our department over 18 months, dividing 60 patients with a single renal or upper ureteric calculus of size ≤2.5 cm into two groups: 30 patients in each group (group 1: tubed PCNL, group 2: tubeless PCNL). The primary outcome was the drop in perioperative hemoglobin level and the number of packed cell transfusions necessary. The secondary outcome included the mean pain score, analgesic requirement, length of hospital stay, time to return to normal activities, and the total cost of the procedure.

Results

The two groups were comparable in age, gender, comorbidities, and stone size. The postoperative hemoglobin level was significantly lower in the tubeless PCNL group (9.56 ± 2.13 gm/dL) compared to the tube PCNL group (11.32 ± 2.35 gm/dL) (p = 0.0037), and two patients in the tubeless group required blood transfusion. The duration of surgery, pain scores, and analgesic requirement were comparable between the two groups. The total procedure cost was significantly lower in the tubeless group (p = 0.0019), and the duration of hospital stay and time to return to daily activities were significantly shorter in the tubeless group (p < 0.0001).

Conclusions

Tubeless PCNL is a safe and effective alternative to conventional tube PCNL, with the advantages of shorter hospital stay, faster recovery, and lower procedure costs. Tube PCNL is associated with less blood loss and the need for transfusions. Patient preferences and bleeding risk should be considered when choosing between the two procedures.

## Introduction

Percutaneous nephrolithotomy (PCNL) is considered to be the procedure of choice for the treatment of renal calculi in the last two decades [1,2]. It was first introduced in 1976, and since then, the operative procedures and the endoscopic equipment underwent many modifications to increase success rates and decrease complications. The standard procedure is to place nephrostomy tubes within the tract of varying caliber and types. This was done to facilitate maximal collecting system drainage, tamponade the access tract, and secure the access in case a second-look PCNL was needed. Multiple studies demonstrate significant morbidity associated with nephrostomy tube following PCNL, mainly postoperative pain that requires significant narcotics and also longer hospital stay.

The idea of the "tubeless" PCNL was born, whereby a nephrostomy tube is not left in place following the percutaneous procedure, but rather, renal drainage is established with an indwelling ureteral stent [3,4]. A large number of studies on tubeless PCNL have been performed, and several previously published systematic reviews have reported its efficacy and safety [5-14]. Several randomized controlled trials (RCTs) have been performed to compare the safety and efficacy of tubeless and standard PCNL, including multiple high-quality RCTs [6,13,15,16]. However, it is important to note that previous RCTs comparing tubeless and tubed PCNL have included a heterogeneous mix of stone sizes and tract sizes. Larger stones typically require larger tract sizes and longer operative times and are associated with an increased risk of bleeding [17]. In contrast, stones that are smaller in size are less complex and represent a subcategory of the overall spectrum of stones. Therefore, conclusions about bleeding risk based on studies with a diverse mix of stone sizes may not accurately reflect the risk of bleeding associated with one particular stone size category. Moderate renal calculi may be associated with lower rates of bleeding due to their smaller size and the relative ease of performing the procedure as compared to large renal calculi [17]. However, it is also possible that these patients may still be at risk for significant blood loss and may benefit from routine nephrostomy tube placement.

Our study aims to add to the available literature on the comparison between tubed and tubeless PCNL by specifically investigating the effect of routine nephrostomy tube placement in patients with moderate renal calculi of size 2.5 cm or less who undergo an uncomplicated PCNL procedure. While previous studies have investigated interventions to reduce blood loss during PCNL, many have not specified whether only uncomplicated cases were included in the analysis. Our study aims to provide a clearer understanding of the effect of routine nephrostomy tube placement on blood loss in a more homogeneous patient population who undergo an uncomplicated PCNL. This will also help to ensure that the results are applicable to routine clinical practice, as uncomplicated cases are more representative of the typical PCNL cases encountered in clinical practice.

## Materials and methods

This was a prospective randomized controlled trial conducted in the Department of Urology at A.J. Institute of Medical Sciences (AJIMS) & Research Centre, Mangalore, India, from February 2017 to August 2018 (18 months). The subjects were divided into two groups of 30 patients each (group 1 including patients undergoing tubed percutaneous nephrolithotomy and group 2 undergoing tubeless PCNL) based on 1:1 sequential randomization to ensure an equal number of patients in both groups. Laboratory parameters such as urine analysis and culture/sensitivity, hemoglobin, electrolytes, blood urea, serum creatinine, and coagulation profile were checked before surgery. Preoperative dual-mode plain computed tomography of kidneys, ureters, and bladder (CT-KUB) was performed in all cases. All adult patients (>18 years) with a single renal calculus of size less than or equal to 2.5 cm in the largest dimension, regardless of calyceal location, were included. Patients with a solitary kidney, uncorrected bleeding diathesis, preoperative eGFR of less than 60 mL/minute/1.73 m^2^, significant intraoperative perforation of the collecting system, significant intraoperative bleeding, congenital renal anomalies, large residual fragments that may require a second-look or staged percutaneous nephrolithotomy, and inability to place a nephrostomy tube due to anatomical reasons were excluded.

Intervention

Under general anesthesia and taking aseptic precautions, a 6-Fr ureteral catheter was introduced into the renal pelvis. The patient was then turned prone, and percutaneous access into the corresponding pelvicalyceal system was achieved under fluoroscopic guidance using an 18-gauge needle. The tract was then dilated using sequential metal dilators up to 26-30 Fr. Nephroscopy was done using either a 20.8-Fr or 26-Fr nephroscope. Stone fragmentation was done using pneumatic or holmium:yttrium-aluminum-garnet (Ho:YAG) laser lithotripsy. All surgeries were performed by a single team of surgeons. Intraoperatively, ureteral abnormalities on retrograde pyelography (RGP), the number of access tracts, the size of Amplatz sheath/sheaths, and the deployment of double J stent were documented. Abdominal ultrasound and/or X-ray KUB were repeated 24 hours after surgery. Complete blood count, serum electrolytes, serum creatinine, and blood urea were repeated on day 1 following surgery.

Outcome measures

For the primary outcome, the drop in hemoglobin in the first 24 hours and the number of units of packed cell transfusions were noted. For the secondary outcome, the mean pain score (using the Wong-Baker FACES Pain rating scale) and analgesic requirement in the postoperative period, length of hospital stay, time to return to normal activities between the two groups, and the total cost of the procedure were calculated. Ultrasonographic evidence of perirenal hematoma/urinoma and the duration of postoperative hospitalization were also noted.

Sample size calculation

The sample size for this study was calculated using a power analysis based on the expected effect size, standard deviation, desired level of significance, and desired power of the study. The primary outcome of interest was the difference in hemoglobin (Hb) levels between the tubed and tubeless percutaneous nephrolithotomy (PCNL) groups.

The formula used for sample size calculation was as follows: n = (2 * (Z_alpha + Z_beta)^2 * σ^2) / Δ^2, where n = sample size per group, Z_alpha = Z-value for the desired level of significance (alpha), Z_beta = Z-value for the desired power of the study (1-beta), σ = standard deviation of the Hb level, and Δ = expected effect size (difference in Hb level).

A hypothetical standard deviation of 1.2 gm/dL for the hemoglobin level was assumed. The desired level of significance (alpha) was set at 0.05, indicating a 5% chance of a type I error, and the desired power of the study (1-beta) was set at 0.80, indicating an 80% chance of detecting a true difference if it exists. The expected effect size (Δ) was assumed to be 1 gm/dL, representing the anticipated difference in Hb levels between the two groups.

Using these values, the sample size per group was calculated, and considering a two-group comparison (tubed and tubeless PCNL), the total sample size was determined to be 56 (28 in each group).

Statistical analysis

Data were coded and entered into Microsoft Excel (Microsoft Corp., Redmond, WA, USA) and analyzed using Statistical Package for the Social Sciences (SPSS) version 16 (IBM SPSS Statistics, Armonk, NY, USA). Demographic data were analyzed using an unpaired t-test or Mann-Whitney U test. Numerical data were analyzed with an unpaired t-test or Mann-Whitney U test based on distribution. Paired parameters were analyzed using a paired t-test. Categorical data were analyzed using a chi-square test.

Informed consent and ethical considerations

Informed consent regarding the nature of the procedure and acceptance for inclusion in the study was taken as per WHO guidelines on patient consent. The study adhered to the ethical principles outlined in the Declaration of Helsinki and obtained approval from the Institutional Ethics Committee of A.J. Institute of Medical Sciences (AJIMS), Mangalore, India (AJEC/REV/16/2017). Patient enrollment only commenced after obtaining ethical clearance.

## Results

A total of 60 patients were enrolled in the study, with 30 in each group. The mean age of the patients was 42.69 ± 10.10 years in the tube PCNL group and 45.36 ± 13.64 years in the tubeless PCNL group (p = 0.067). There were 21 males and nine females in the tube PCNL group and 20 males and 10 females in the tubeless PCNL group (p = 0.078). The comorbidities were comparable between the two groups (p = 0.09). The mean stone size was 2.33 ± 0.42 cm^2^ in both groups (p = 0.97). The preoperative hemoglobin level was also comparable between the two groups (p = 0.26). The postoperative hemoglobin level was significantly lower in the tubeless PCNL group (9.56 ± 2.13 gm/dL) compared to the tube PCNL group (11.32 ± 2.35 gm/dL) (p = 0.0037). Two patients in the tubeless PCNL group required a single unit of packed cell transfusion each, whereas none in the tube PCNL group required transfusion (p = 0.001).

**Figure 1:**
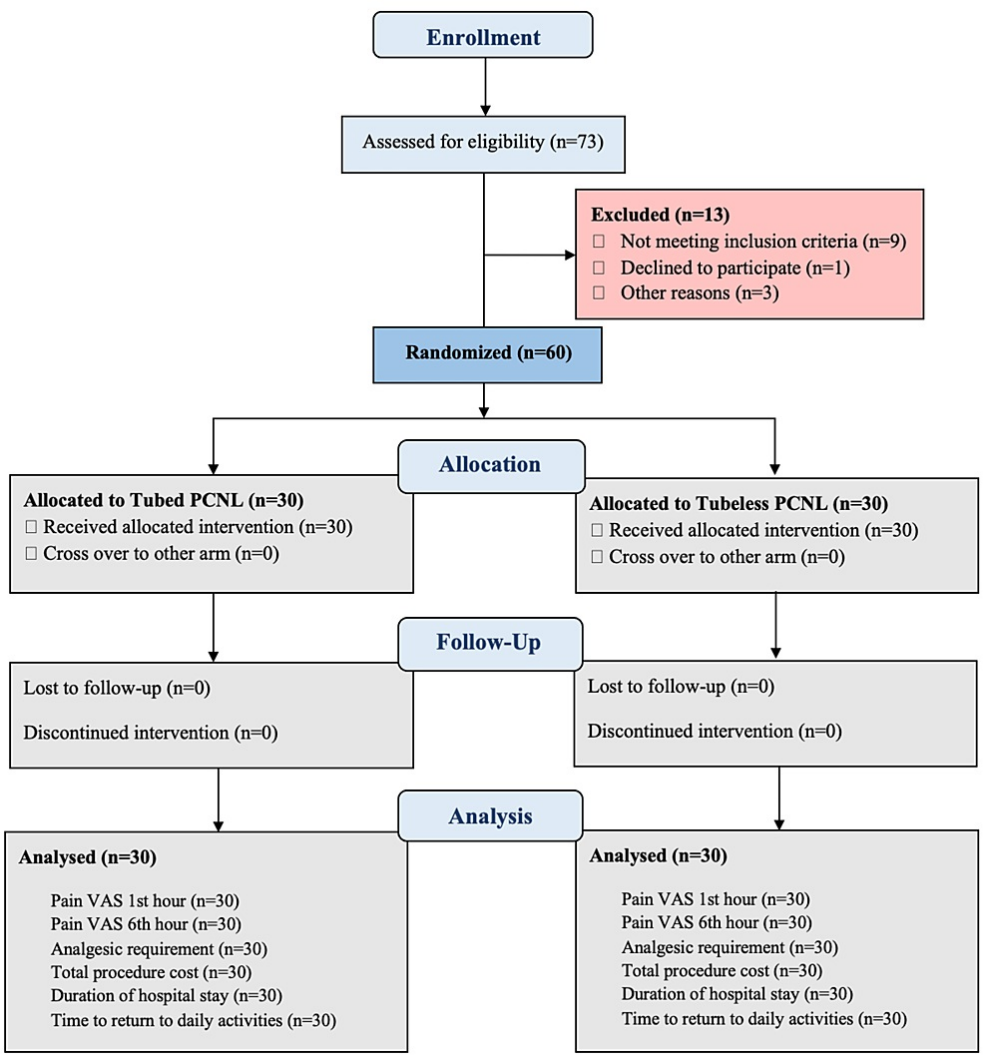
CONSORT diagram of the study showing comparative assessment of both groups. PCNL: percutaneous nephrolithotomy, VAS: visual analog scale

The duration of surgery was comparable between the two groups (p = 0.84). The pain score, as measured using the visual analog scale (VAS), was also comparable between the two groups at one hour (6.30 ± 1.12 versus 6.23 ± 1.14, p = 0.82) and six hours (4.77 ± 0.68 versus 4.40 ± 0.67, p = 0.40) after the procedure. The analgesic requirement, as measured by the intravenous tramadol dosage, was also comparable between the two groups (60.90 ± 8.09 versus 59.30 ± 6.89, p = 0.41). However, the total procedure cost was significantly lower in the tubeless PCNL group (Rs. 28,259 ± 3,712) compared to the tube PCNL group (Rs. 31,705 ± 4,476) (p = 0.0019). The duration of hospital stay was significantly shorter in the tubeless PCNL group (2.57 ± 0.73 days) compared to the tube PCNL group (3.47 ± 0.68 days) (p < 0.0001). The time to return to daily activities was also significantly shorter in the tubeless PCNL group (5.77 ± 1.01 days) compared to the tube PCNL group (7.17 ± 1.26 days) (p < 0.0001). Figure 1 represents the CONSORT diagram of the study showing a comparative assessment of both groups. Table 1 presents a comparative description of baseline variables and outcome measures of both the studied groups.

**Table 1:** Comparative description of the baseline variables and outcome measures in both groups. PCNL: percutaneous nephrolithotomy, Hb: hemoglobin, VAS: visual analog score, i.v.: intravenous, mg: milligrams, INR: Indian Rupee

Characteristics		Tubed PCNL	Tubeless PCNL	p-value
Number of patients (number)		30	30	
Mean age (years)		42.69 ± 10.10	45.36 ± 13.64	0.067
Gender	Male	21	20	0.078
	Female	9	10	
Comorbidities		11	8	0.09
Stone size (cm)		2.33 ± 0.42	2.33 ± 0.45	0.97
Preoperative Hb (gm/dL)		11.32 ± 2.36	10.50 ± 2.78	0.26
Postoperative Hb (gm/dL)		11.32 ± 2.35	9.56 ± 2.13	0.0037
Number of units of packed cell transfusion		0	2	0.001
Duration of surgery		54.73 ± 10.66	55.30 ± 10.42	0.84
Pain VAS first hour		6.30 ± 1.12	6.23 ± 1.14	0.82
Pain VAS sixth hour		4.77 ± 0.68	4.40 ± 0.67	0.40
Analgesic requirement (i.v. tramadol in mg)		60.90 ± 8.09	59.30 ± 6.89	0.41
Total procedure cost (INR)		31,705 ± 4,476	28,259 ± 3,712	0.0019
Duration of hospital stay (days)		3.47 ± 0.68	2.57 ± 0.73	<0.0001
Time to return to daily activities (days)		7.17 ± 1.26	5.77 ± 1.01	<0.0001

## Discussion

The results of this study comparing tubeless percutaneous nephrolithotomy (PCNL) and conventional tube PCNL indicate that tubeless PCNL is a safe and effective alternative to conventional PCNL. The study found no significant difference between the two techniques in terms of gender, comorbidities, and stone size. However, the tubeless PCNL group had a higher mean age compared to the conventional tube PCNL group.

In clinical practice, the size and complexity of a kidney stone can greatly influence the choice of treatment and the risk of complications [17]. By restricting our study population to patients with moderate stones measuring 2.5 cm or less, we aimed to minimize the confounding effects of stone size and complexity on operative outcomes and the risk of complications. By doing so, we can isolate the specific effects of tubeless versus tubed PCNL on this patient population. Furthermore, by focusing on uncomplicated operative procedures, we can also minimize the confounding effects of comorbidities and other factors that may influence the risk of complications. This will help us to obtain a more accurate understanding of the true risks and benefits associated with tubeless versus tubed PCNL in a specific patient cohort. Overall, by carefully selecting our study population and controlling for potential confounders, we aimed to gain a more precise understanding of the optimal approach for treating patients with specifically smaller kidney stones.

One of the primary outcomes of this study was postoperative bleeding, which was significantly higher in the tubeless PCNL group than in the tube PCNL group. The tubeless PCNL group also required more blood transfusions than the tube PCNL group. However, the risk of bleeding can be minimized with careful patient selection, appropriate patient preparation, and meticulous surgical technique. Hence, the use of the tubeless PCNL technique should be cautiously decided after considering the potential risks and benefits.

One of the other significant findings of this study was the reduced duration of hospital stay in the tubeless PCNL group compared to the conventional tube PCNL group. Patients who underwent tubeless PCNL had a mean hospital stay of 2.57 ± 0.73 days, while patients who underwent conventional tube PCNL had a mean hospital stay of 3.47 ± 0.68 days (p < 0.0001). These results correlate well with the results of previous studies [5-8]. The shorter duration of hospital stay in the tubeless PCNL group is attributable to a faster recovery and return to daily activities. The study found that patients who underwent tubeless PCNL also had a significantly shorter time to return to daily activities compared to those who underwent conventional tube PCNL (5.77 ± 1.01 versus 7.17 ± 1.26 days, p < 0.0001).

The study found no significant difference between the two techniques in terms of pain experienced by patients in the first and sixth hours after surgery, as well as the analgesic requirement. Both groups had similar pain scores and required similar doses of intravenous tramadol.

The reduced duration of hospital stay and faster recovery in the tubeless PCNL group can have several advantages, including reduced healthcare costs and overall patient satisfaction. The study found that the total procedure cost was significantly lower in the tubeless PCNL group compared to the conventional tube PCNL group (28,259 ± 3,712 versus 31,705 ± 4,476, p = 0.0019). This can be attributed to the reduced duration of hospital stay in the tubeless PCNL group and the cost of a nephrostomy tube. In resource-limited settings, the use of healthcare resources must be optimized to provide cost-effective care to patients with kidney stones. Current judgments about which interventions and programs are cost-effective are often aspirational and do not reflect the reality of resource constraints [18]. The study findings provide evidence that careful patient selection and the appropriate use of the tubeless PCNL technique can help optimize healthcare resources and provide cost-effective care to patients. However, it is important to note that the decision to perform tube or tubeless PCNL should be based on individual patient characteristics, preferences, and potential risks and benefits. Patients who are at high risk of bleeding or who require longer postoperative monitoring and care may be better suited for the tube PCNL approach, which may result in a longer hospital stay and higher healthcare costs but may also be safer for these patients. On the other hand, patients who can be carefully selected for the tubeless technique and prioritize a shorter hospital stay and quicker return to daily activities may be good candidates for this approach.

The strength of our study lies in its measurement of multiple outcomes, including clinical, economic, and patient-reported outcomes, providing a comprehensive assessment of the benefits and risks of the two procedures. This approach allows healthcare providers to have a more complete understanding of the effects of the procedures, beyond just clinical outcomes, and make more informed decisions about which procedure to use for their patients. Additionally, the inclusion of economic and patient-reported outcomes provides a more patient-centered approach to the assessment of the procedures, which are particularly relevant in resource-limited settings.

However, the study acknowledges several limitations that should be considered when interpreting the results. One of the main limitations of the study is the relatively small sample size, with only 30 patients in each group. This small sample size may limit the statistical power of the study, making it more difficult to detect statistically significant differences between the groups, especially for outcomes that have a low incidence rate. Additionally, the study was conducted at a single center, which may limit the generalizability of the results to other institutions and patient populations.

## Conclusions

In conclusion, this study suggests that tubeless PCNL is a safe and effective alternative to conventional tube PCNL. Tubed PCNL is associated with lesser operative blood loss and the need for blood transfusions. Tubeless PCNL is associated with a shorter duration of hospital stay, faster recovery, and lower total procedure cost. These advantages of tubeless PCNL can lead to improved patient satisfaction, reduced healthcare costs, and improved clinical outcomes. Overall, tubeless PCNL may be a preferred option for patients who prioritize cost-effectiveness and faster recovery, while tubed PCNL may be preferred in patients who are at a higher risk of bleeding.
